# Development and validation of assistance needs assessment scale of the home-dwelling older adults

**DOI:** 10.1186/s12877-025-06539-8

**Published:** 2025-10-31

**Authors:** Lei Huang, Weihong Yang, Jiaqi Yang, Panpan Jing, Yuqing Ye, Yifei Zhao, Chuanwu Lyu, Xianghan Kong, Lina Wang, Peng Wang, Yan Lin, Rui Chen, Yue Yao, Yang Li

**Affiliations:** 1 Nursing department, The First Affiliated Hospital of Henan Medical University, Xinxiang, China; 2 School of Nursing, Henan Medical University, Xinxiang, China; 3 Department of Clinical Laboratory, Weihui People’s Hospital, Xinxiang, China; 4 Department of Infection Control, Luoyang Orthopedic Hospital of Henan Province, Orthopedic Hospital of Henan Province, Zhengzhou, China; 5 Second Department of Spinal Pain, Luoyang Orthopedic-Traumatological Hospital of Henan Province (Henan Provincial Orthopedic Hospital), Luoyang, China

**Keywords:** Older adults, Home-dwelling, Assistance, Need, Scale

## Abstract

**Background:**

With the acceleration of population aging, identifying and addressing the unmet assistance needs of home-dwelling older adults has become increasingly important. This study aimed to develop and validate a reliable and practical assessment tool to identify such needs.

**Methods:**

A descriptive qualitative study was conducted using semi-structured interviews with home-dwelling older adults in two communities in Wuhan, China. Based on the qualitative findings and literature review, an initial item pool was developed, followed by expert consultation to construct a draft scale. A cross-sectional survey was then conducted in communities across four central Chinese cities. Item analysis and psychometric testing were performed to finalize the scale.

**Results:**

The finalized scale consists of 27 items across four dimensions: daily assistance, health maintenance, visitation and communication, and social interaction. A total of 380 older adults participated in the study, with 170 used for item screening and 210 for validation. Confirmatory factor analysis showed good model fit (CFI= 0.951, TLI = 0.946, RMSEA = 0.068). Standardized factor loadings ranged from 0.67 to 0.94. The scale demonstrated excellent internal consistency, with Cronbach’s α ranging from 0.943 to 0.968, and met all reliability and validity standards.

**Discussion:**

The developed scale has sound psychometric properties and is a reliable tool for assessing the unpaid assistance needs of home-dwelling older adults. It holds promise for guiding community service planning, public welfare resource allocation, and policy development in the context of population aging.

## Introduction

With the accelerating pace of global population aging, the aging crisis has become a major societal challenge faced by countries worldwide. According to United Nations data, the global population aged 60 and above is projected to reach 1.4 billion by 2030 and 2.1 billion by 2050 [1]. China, as the world’s most populous developing country, is experiencing particularly rapid aging due to its socioeconomic development. By the end of 2023, approximately 297 million people in China were aged 60 and above, accounting for 21.1% of the total population [2]. This rapidly growing elderly population places increasing pressure on China’s social security and healthcare systems, further widening the gap between the supply and demand for care services of older adults [3, 4].

China currently promotes the “9073” care model of older adults, in which approximately 90% of older adults receive care at home, 7% rely on community-based care, and only 3% live in institutional settings [5]. Home-based care remains the dominant form of care of older adults in China. However, with the ongoing transformation of family structures toward smaller, nuclear, and empty-nest households, traditional family-based care functions are weakening. As a result, many home-dwelling older adults lack adequate family support due to factors such as living alone or having adult children occupied with work responsibilities [6, 7]. Recent studies show that the satisfaction rate of perceived care needs of older adults among community-dwelling older adults in China ranges from only 9.1% to 45.0%. In contrast, the reported demand for services is as high as 64.9% to 81.2% [8]. This indicates a significant service gap that requires urgent attention. Other studies have found that long-term unmet needs can severely compromise the physical and mental health of older adults, and in some cases, even endanger their lives [9, 10]. Therefore, it is imperative to expand the availability of community-based care resources of older adults for this population [11].

In recent years, China has actively promoted the development of volunteer services and neighborhood mutual assistance as a means to enhance the social support system for home-based care of older adults. The “14th Five-Year Plan for the Development of Aging Services and Elderly Care System” clearly advocates for the establishment of an integrated model involving social workers, neighbors, volunteers, and physicians to provide psychosocial support services to older adults with special difficulties [12]. President Xi Jinping has also emphasized the importance of strengthening public participation mechanisms and developing volunteer-based care services of older adults to address the country’s demographic aging [13]. Given these policy directions, there is an urgent need for a dedicated assessment tool to evaluate the assistance needs of home-dwelling older adults within informal and non-paid support systems, including volunteers, neighbors, and community mutual-aid networks, rather than through paid formal providers. Such a tool is essential for scientifically identifying and assessing actual support needs, thereby enabling communities to deliver more targeted and effective services.

Although several tools have been developed to assess the needs of home-dwelling older adults, most focus on medical care, functional support, or general care needs of older adults. These tools often do not specifically address unpaid assistance needs within informal care systems [14–19]. For example, Shih et al. [16] developed the Health Needs Instrument (HNI), which uses binary “yes/no” responses to evaluate older adults’ needs across five dimensions: tangible assistance, emotional support, medical help, access to health information, and participation in decision-making. However, the tool does not clearly distinguish between formal and informal support sources. Chatterjee et al. [17] developed the Integrated Care Tool-Brief (ICT-BRIEF), which includes 30 items covering physical health, functional status, geriatric syndromes, psychological well-being, and social domains. Although useful for preliminary screening and care planning, the tool is still situated within a broader health assessment framework. Some studies have touched upon volunteer service needs, but these tools are not specifically designed to assess the unpaid assistance needs of home-dwelling older adults. For instance, Zhao et al. [18] conducted a survey using a self-developed questionnaire to assess older adults’ needs for volunteer services, but the tool used binary response options and included institutionalized individuals, limiting its representativeness. Wilkinson-Meyers et al. [19] developed a questionnaire to assess support needs across seven domains (e.g., personal care, household chores, meal preparation, shopping, and transportation), evaluating assistance provided by family, friends, or paid/unpaid service providers. While partially addressing unpaid support, this instrument lacks a clear focus on home-dwelling older adults and includes paid assistance.

In summary, current assessment tools generally lack targeted focus on the concept of unpaid assistance needs, particularly in identifying the extent to which older adults expect to receive support from non-family, unpaid social resources. Therefore, it is essential to develop a scientific, systematic, and culturally adapted instrument specifically designed to assess such needs in the context of informal caregiving. This study aims to develop an assessment scale for the unpaid assistance needs of home-dwelling older adults in China by integrating literature review, qualitative interviews, and expert consultation to construct a comprehensive evaluation framework. The results will provide key stakeholders—such as communities, healthcare institutions, and volunteer organizations—with more accurate information to better match and allocate support resources, ultimately improving the quality of life for older adults [20, 21]. Furthermore, the scale will serve as a reference for other countries and regions facing similar challenges, contributing to the sustainable response to population aging and advancing the global strategy for healthy aging.

## Method

This study adhered to the established principles and methodologies for scale development, combining literature review, qualitative research, and expert consultation. By employing a qualitative descriptive design, the study aimed to minimize researcher bias, allowing findings to emerge directly from the data [22, 23]. An initial item pool was created based on the literature review and qualitative research findings. Subsequently, expert consultations were conducted to formulate a preliminary version of the Home-Dwelling Older Adults Assistance Needs Assessment Scale. A cross-sectional survey was conducted using convenience sampling in four communities across four cities in central China (Wuhan, Ezhou, Zhengzhou, and Xinxiang). The selection of these sites was guided by several considerations, including their alignment with national development strategies for the central region, geographic accessibility, feasibility of collaboration with local institutions, and the research team’s established connections with community organizations. Based on the data collected, the scale was further refined and finalized through item screening as well as reliability and validity analyses. Participant Inclusion and Exclusion Criteria Inclusion criteria were: (1) aged 60 years or older; (2) currently residing in an urban community in China and having lived there for more than one year; (3) informed of the research purpose and voluntarily participating. Exclusion criteria: individuals with communication barriers.

### Stage 1: item pool generation

A comprehensive literature search was conducted using keywords such as “older adult,” “home-dwelling,” “community-dwelling,” “assistance,” “need,” “questionnaire,” and “scale” across multiple databases (PubMed, EMBASE, CINAHL Complete, Scopus, Web of Science, CNKI, Wanfang, VIP). This search informed the development of the qualitative interview guide and the questionnaire item pool.

A descriptive qualitative research design was employed. Purposive sampling (including criterion-based and maximum variation sampling) was used to recruit home-dwelling older adults from two geographically distant communities in Wuhan, China. Maximum variation sampling ensured representation of older adults with diverse life difficulties (e.g., living alone, chronic illnesses) and various age groups. Data collection continued until saturation was achieved. Textual data were managed and analyzed using NVivo 11.0 Plus software, employing traditional content analysis methods [24]. The interview guide is detailed in Appendix 1. Findings from this qualitative study have been presented in a previously published study [25].

The initial structure and content of the scale were determined using thematic analysis based on the literature review and semi-structured interviews [26].

### Stage 2: Delphi expert consultation

The Delphi method was implemented with two rounds of expert consultation to refine the preliminary scale by adding, deleting, and modifying items. The number of experts is determined based on the scale of the research project, and typically ranges from 15 to 50 [27]; this study invited 17 experts. Detailed information about the Delphi panel, including their demographic and professional characteristics, is provided in Appendix 2. The first round of expert consultation comprised four parts: (1) introduction and instructions detailing the purpose and significance of the scale; (2) expert consultation form evaluating item relevance (4-point Likert scale) and importance (5-point Likert scale) [28]; (3) expert demographic questionnaire collecting institutional affiliation, gender, age, contact information, educational background, professional title, expertise, and years of experience; (4) authority questionnaire assessing experts’ familiarity and judgment basis regarding the scale content. The second round also included four similar parts with additional descriptions of the scale’s revisions.

Both consultation rounds were conducted online via email or WeChat, requiring responses within two weeks. After the first round, the research team collected and analyzed the data, revised the items accordingly, and sent a second-round questionnaire to the same experts for reassessment. Consultation ceased once expert consensus was achieved.

Data analysis was conducted by two researchers using SPSS 22.0 software. Expert opinions and statistical analyses guided final decisions on item inclusion, deletion, or modification, ultimately producing the finalized scale. Evaluation metrics included: (1) response rate (> 70% indicating high engagement); (2) authority coefficient (Cr ≥ 0.70 indicating high expert authority); (3) opinion concentration (mean scores ≥ 4 indicating high consensus) [29]; (4) coordination of opinions (coefficient of variation Cv < 0.25 indicating minimal disagreement) [30]; and (5) content validity indices (CVI: item-level CVI ≥ 0.78, scale-level CVI ≥ 0.90) [31].

### Stage 3: psychometric testing

The required sample size for scale development considered both exploratory factor analysis (EFA) and confirmatory factor analysis (CFA). Generally, the EFA sample size should be 5–10 times the total number of scale items [32]. Given the preliminary scale had 33 items across 4 dimensions, the sample size required for EFA was at least 165 cases. The CFA sample size needed to be no less than 200 and larger than the EFA sample size [33]. Accounting for a 10% rate of invalid responses, the total sample size required was at least 406.

The survey consisted of two instruments: (1) a demographic questionnaire developed by the researchers to collect sociodemographic information from participants, including gender, age, education level, religious belief, previous occupation, and marital status; (2) the preliminary version of the Home-Dwelling Older Adults Assistance Needs Assessment Scale, developed by the researchers, comprising 33 items in four dimensions: life assistance (10 items), health maintenance (9 items), visiting and communication (8 items), and social interaction (6 items). Items were rated on a 5-point Likert scale ranging from 1 (not needed at all) to 5 (very much needed), with total scores ranging from 33 to 165. Higher scores indicated greater overall needs for volunteer services. A standardized instruction was provided at the beginning of the questionnaire to ensure consistent understanding. The instruction specified that the term “someone” referred to volunteers, neighbors, or other informal helpers providing unpaid support, rather than paid professional caregivers or service providers. This instruction was presented before the items to guide respondents in completing the scale. (4) the Activity of Daily Living Scale (ADL), originally developed by Mahoney and Barthel in 1965 [34], is widely used to assess the self-care ability of older adults or individuals with chronic diseases in performing everyday activities. It consists of 10 items, each scored using different ranges depending on the task. For example, some items are rated 0, 5, or 10, whereas others are rated 0, 5, 10, or 15. The total score ranges from 0 to 100, with higher scores indicating better independence in daily functioning. In the present study, the Cronbach’s alpha of the ADL scale was 0.933, suggesting excellent internal consistency. (5) the Patient Health Questionnaire (PHQ-9), developed by Kroenke and colleagues [35] based on the diagnostic criteria for depressive disorders in the DSM-IV, is commonly used for screening, diagnosis, and monitoring of depression in adults. It includes 9 items, each rated on a 4-point Likert scale from 0 (not at all) to 3 (nearly every day). The total score ranges from 0 to 27, with higher scores reflecting more severe depressive symptoms. In this study, the PHQ-9 demonstrated excellent internal reliability, with a Cronbach’s alpha of 0.939.

All researchers received standardized training to clarify study objectives and protocols. Before initiating data collection, the researchers contacted community leaders or management authorities to obtain consent and ensure smooth survey administration. A preliminary survey involving 30 older adults from the communities was conducted to refine questionnaire wording. Subsequently, the researchers obtained contact and residence information of potential participants through community management offices, contacted the older adults or their family members by phone, and verified eligibility criteria. Upon obtaining informed consent, researchers visited participants at their homes, accompanied by community staff, to administer the survey. During survey completion, researchers supervised and provided necessary explanations to ensure data quality. Completed surveys were immediately checked, and any incomplete or incorrectly filled questionnaires were returned to participants for correction. The survey adhered strictly to anonymity and confidentiality.

Statistical analysis was conducted using SPSS 26.0 and AMOS 24.0 software. (1) Descriptive analysis: normally distributed continuous data were presented as mean ± SD, and skewed data as median [M (P25, P75)]; categorical data were presented as percentages (%) and frequencies (n). (2) Item screening methods included: (a) critical ratio method: evaluating item discrimination, with items having p-values > 0.05 considered for deletion [33]; (b) correlation coefficient method: assessing correlations between individual items and total scale scores, with items having correlation coefficients < 0.4 or p-values > 0.05 considered for deletion [33]; (c) Cronbach’s alpha method: examining changes in the overall Cronbach’s alpha upon item deletion, with increases indicating poor homogeneity and thus suggesting deletion [33]; (d) exploratory factor analysis: after confirming suitability through Bartlett’s test (*p* < 0.05) and Kaiser-Meyer-Olkin (KMO) measure (> 0.60), factors with eigenvalues > 1 explaining at least 50% cumulative variance were extracted using principal component analysis with varimax rotation, and items with factor loadings < 0.40 were considered for deletion [36]. (3) Validity analyses: (a) construct validity assessed using CFA with fit indices (CFI > 0.90, TLI > 0.90, RMSEA < 0.08, χ²/df < 3, NFI > 0.90, IFI > 0.90), and factor loadings ideally between 0.71 and 0.95 [34]; (b) convergent validity evaluated using average variance extracted (AVE ≥ 0.50) and composite reliability (CR ≥ 0.60) [37]; (c) predictive validity preliminarily examined by testing correlations between scale scores and external indicators [38]. (4) Reliability analyses included internal consistency (Cronbach’s alpha > 0.80) and split-half reliability (> 0.80) [39]. (5) Acceptability indicators: response rate and valid response rate ≥ 85%, and an average completion time of 15–20 min recommended [40].

## Results

### Item pool development

The initial item pool for the Home-Dwelling Older Adults Assistance Needs Assessment Scale comprised 37 items across five dimensions: daily care (9 items), medical care (10 items), psychological comfort (10 items), cultural engagement (4 items), and social interaction (4 items). Items were rated using a 5-point Likert scale (1 = not needed at all, 2 = not really needed, 3 = uncertain, 4 = needed, 5 = very much needed) (Appendix 3).

### Qualitative evaluation

In the first Delphi expert consultation round, 17 experts were invited, and 15 valid responses were collected. Experts were from seven provinces (Hubei, Henan, Jiangsu, Sichuan, Chongqing, Guangdong, Fujian), representing various fields including geriatric nursing, community nursing, social security, clinical nursing, psychological nursing, and nursing management. The mean expert age was 51.33 ± 7.08 years, with an average of 27.93 ± 11.11 years of relevant experience. Further details of the expert consultation are provided in Appendix 2. The response rates for the first and second rounds were 88.2% (15 out of 17) and 100.0% (15 out of 15), respectively, indicating high expert engagement (> 70%). The authority coefficient (Cr) was consistently high at 0.86 across both rounds. Mean importance ratings ranged from 4.13 to 4.60 in the first round and 4.00–4.93 in the second round, demonstrating a high concentration of expert opinions (Appendices 4 and 5). Coefficients of variation ranged from 0.14 to 0.23 (round 1) to 0.05–0.23 (round 2), indicating strong expert consensus (Appendices 4 and 5). Item-level content validity indices (I-CVI) improved from 0.47 to 0.93 (round 1) to 0.80–1.00 (round 2), with the scale-level content validity index (S-CVI) increasing from 0.84 to 0.96. After two rounds of consultation, all items met the required standards, indicating that the content validity aligns with the requirements for scale development. Detailed results are presented in Appendix 6 and 7. In addition, during the first round of expert consultation, a total of 31 revision suggestions were collected, of which 25 were adopted or thoroughly considered. Based on expert recommendations, corresponding items in the scale were deleted, modified, or added. In the second round, 6 suggestions were received, all of which were accepted or fully taken into account. The scale items were revised accordingly based on these expert inputs. The detailed revision process is presented in Appendix 8.

### Quantitative evaluation

A total of 440 questionnaires were distributed, with 394 returned (89.5% response rate). After excluding invalid responses, 380 valid questionnaires were analyzed (86.4% valid response rate). Participants’ ages ranged from 60 to 98 years, with a median age of 72 (66, 80) years (Appendix 9). From this dataset, 170 randomly selected cases were used for item analysis, reliability testing, and exploratory factor analysis (EFA), while the remaining 210 cases underwent confirmatory factor analysis (CFA).

#### Item selection

Using the critical ratio method, 170 cases were divided into high- and low-scoring groups (top and bottom 27%, *n* = 46 each). Independent-sample t-tests showed significant differences between the two groups, with t-values ranging from 7.307 to 24.318 (all *p* < 0.001), indicating excellent item discrimination (Table 1). In the correlation analysis, item-total correlation coefficients ranged from 0.548 to 0.864, demonstrating high internal consistency (Table 2), and all items were retained based on this result. According to the Cronbach’s alpha method, the overall Cronbach’s alpha was 0.974. Deletion of any single item did not significantly increase the alpha value. The corrected item-total correlations ranged from 0.523 to 0.852, and the dimension-level Cronbach’s alpha coefficients remained similarly stable, ranging from 0.704 to 0.887, further confirming strong internal consistency (Appendices 8 − 1 and 8 − 2). In the exploratory factor analysis, the Kaiser-Meyer-Olkin (KMO) measure was 0.929, and Bartlett’s test of sphericity was significant (χ² = 5409.043, *p* < 0.001), indicating the dataset’s suitability for factor analysis. Four factors with eigenvalues greater than 1 were extracted, explaining a cumulative variance of 79.6%. Six items (a7, a8, b8, b9, c2, c3) showed ambiguous loadings on multiple factors and were removed following team discussion. The final factor loadings for retained items ranged from 0.560 to 0.909.

**Table 1:** Critical ratio analysis results

Dimension	Item	Levene’s Test for Homogeneity of Variance		Equal Variance	t	*P*
		F	*P*			
Daily Living Assistance	a1	1.335	0.251	Yes	16.990	<0.001
	a2	0.461	0.499	Yes	15.057	<0.001
	a3	30.704	<0.001	No	11.105	<0.001
	a4	39.549	<0.001	No	9.283	<0.001
	a5	0.005	0.942	Yes	17.796	<0.001
	a6	4.387	0.039	No	20.857	<0.001
	a7	8.194	0.005	No	12.378	<0.001
	a8	3.611	0.061	Yes	24.318	<0.001
	a9	5.835	0.018	No	14.850	<0.001
	a10	1.208	0.275	Yes	12.559	<0.001
Health Maintenance	b1	23.059	<0.001	No	11.636	<0.001
	b2	43.545	<0.001	No	11.172	<0.001
	b3	40.959	<0.001	No	11.566	<0.001
	b4	20.858	<0.001	No	13.294	<0.001
	b5	5.176	0.025	No	14.746	<0.001
	b6	59.538	<0.001	No	9.544	<0.001
	b7	7.498	0.007	No	14.525	<0.001
	b8	20.137	<0.001	No	10.687	<0.001
	b9	16.307	<0.001	No	11.001	<0.001
Visiting and Communication	c1	1.386	0.242	Yes	15.202	<0.001
	c2	0.132	0.717	Yes	11.872	<0.001
	c3	0.223	0.638	Yes	12.732	<0.001
	c4	10.074	0.002	No	12.647	<0.001
	c5	7.527	0.007	No	11.530	<0.001
	c6	8.646	0.004	No	12.457	<0.001
	c7	12.467	0.001	No	9.620	<0.001
	c8	2.248	0.137	Yes	14.318	<0.001
Social Engagement	d1	11.033	0.001	No	8.826	<0.001
	d2	14.075	<0.001	No	8.183	<0.001
	d3	21.372	<0.001	No	7.471	<0.001
	d4	13.546	<0.001	No	7.307	<0.001
	d5	6.901	0.010	No	8.491	<0.001
	d6	12.987	0.001	No	8.236	<0.001

**Table 2:** Correlation analysis between scale items and total scores

Item	*r*	*P*	Item	*r*	*P*	Item	*r*	*P*
a1	0.789	<0.001	b2	0.739	<0.001	c4	0.743	<0.001
a2	0.761	<0.001	b3	0.759	<0.001	c5	0.736	<0.001
a3	0.635	<0.001	b4	0.788	<0.001	c6	0.761	<0.001
a4	0.623	<0.001	b5	0.812	<0.001	c7	0.678	<0.001
a5	0.821	<0.001	b6	0.720	<0.001	c8	0.775	<0.001
a6	0.796	<0.001	b7	0.803	<0.001	d1	0.562	<0.001
a7	0.750	<0.001	b8	0.766	<0.001	d2	0.548	<0.001
a8	0.864	<0.001	b9	0.774	<0.001	d3	0.548	<0.001
a9	0.772	<0.001	c1	0.803	<0.001	d4	0.567	<0.001
a10	0.698	<0.001	c2	0.758	<0.001	d5	0.592	<0.001
b1	0.744	<0.001	c3	0.761	<0.001	d6	0.634	<0.001

#### Validity analysis

**Fig. 1:**
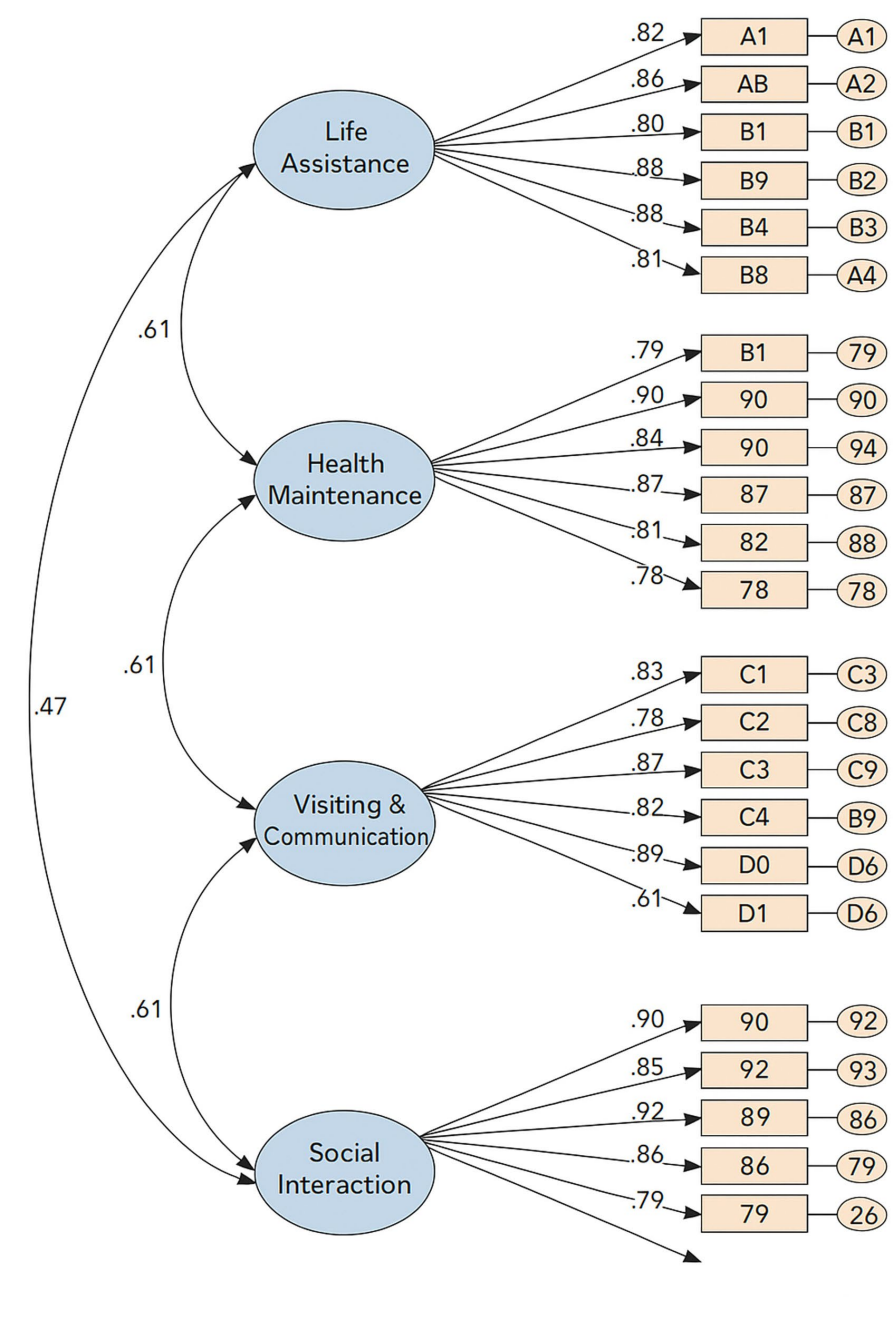
Confirmatory Factor Analysis Model

Construct validity was supported by confirmatory factor analysis (CFA), which demonstrated good model fit indices (CFI = 0.951, TLI = 0.946, RMSEA = 0.068, χ²/df = 1.971, NFI = 0.906, IFI = 0.952), indicating that the hypothesized model aligned well with the observed data (Fig. 1). In terms of average variance extracted (AVE) and composite reliability (CR), standardized factor loadings ranged from 0.67 to 0.94. The CR values ranged from 0.945 to 0.967, and AVE values ranged from 0.685 to 0.832, confirming strong convergent validity of the scale (Table 3). Regarding predictive validity, Spearman correlation analysis showed that scale scores were moderately and positively correlated with depressive symptoms (*r* = 0.384, *p* < 0.001) and weakly and negatively correlated with activities of daily living (*r* = − 0.170, *p* = 0.013), consistent with theoretical expectations.

**Table 3:** Average variance extracted and composite reliability

Item	Dimension	Standardized Factor Loading	Significance	Composite Reliability	Average Variance Extracted
a1	Daily Living Assistance	0.82		0.945	0.685
a2		0.86	***		
a3		0.86	***		
a4		0.88	***		
a5		0.84	***		
a6		0.87	***		
a9		0.67	***		
a10		0.80	***		
b1	Health Maintenance	0.88		0.963	0.788
b2		0.88	***		
b3		0.92	***		
b4		0.90	***		
b5		0.91	***		
b6		0.84	***		
b7		0.88	***		
c1	Visiting and Communication	0.83		0.951	0.764
c4		0.89	***		
c5		0.83	***		
c6		0.92	***		
c7		0.87	***		
c8		0.90	***		
d1	Social Engagement	0.90		0.967	0.832
d2		0.92	***		
d3		0.93	***		
d4		0.94	***		
d5		0.89	***		
d6		0.89	***		

***indicates *P*<0.001

#### Reliability analysis

The finalized scale’s Cronbach’s alpha was 0.968 overall and ranged from 0.943 to 0.967 across dimensions. Split-half reliability was 0.969 overall, with dimensions ranging from 0.914 to 0.948, indicating excellent reliability.

#### Acceptability analysis

The questionnaire achieved a response rate of 89.5% and a valid response rate of 86.4%, reflecting strong acceptability. The finalized Home-Dwelling Older Adults Assistance Needs Assessment Scale comprises 27 items across four dimensions: life assistance (8 items), health maintenance (7 items), visiting and communication (6 items), and social interaction (6 items) (Table 4).

**Table 4:** Finalized scale items for Home-Dwelling older adults assistance needs assessment (Instruction: please read the following items and indicate how much you would need such assistance in your daily life. In this questionnaire, “someone” specifically refers to volunteers, neighbors, or other informal helpers providing unpaid support, rather than paid professional caregivers or service providers.)

Dimensions and Items	Item Content
Dimension 1	Daily Living Assistance
a1	How would you feel if someone could assist you with grocery shopping or other purchases?
a2	How would you feel if someone could deliver meals to you and improve your diet?
a3	How would you feel if someone could help you maintain personal hygiene, such as washing, bathing, or changing clothes?
a4	How would you feel if someone could assist you with indoor mobility?
a5	How would you feel if someone could accompany you for outdoor walks?
a6	How would you feel if someone could help you with house cleaning?
a9	How would you feel if someone could teach you how to use a smartphone or access the internet to enrich your life and increase enjoyment?
a10	How would you feel if someone could accompany you to the bank to handle deposits, withdrawals, or financial matters?
Dimension 2	Health Maintenance
b1	How would you feel if someone could come to your home to check your blood pressure, blood sugar, and conduct other physical examinations?
b2	How would you feel if someone could provide you with medication guidance?
b3	How would you feel if someone could offer you health care and disease prevention advice?
b4	How would you feel if someone could give you fall prevention guidance?
b5	How would you feel if someone could accompany you to hospital visits or health check-ups?
b6	How would you feel if someone could respond promptly and provide help when you actively seek assistance?
b7	How would you feel if someone could guide and accompany you in rehabilitation and functional exercises?
Dimension 3	Visiting and Communication
c1	How would you feel if someone could chat with you?
c4	How would you feel if someone could listen to your past experiences and praise or affirm your achievements?
c5	How would you feel if someone could celebrate your birthday?
c6	How would you feel if someone could recognize your negative emotions during conversations and offer appropriate advice?
c7	How would you feel if someone made you feel respected and appreciated during visits?
c8	How would you feel if someone could show concern for your family and help ease your worries about their difficulties?
Dimension 4	Social Engagement
d1	How would you feel if someone could register or help you join social activities?
d2	How would you feel if someone could accompany you to social activities?
d3	How would you feel if someone could organize social or group activities for older adults?
d4	How would you feel if someone could help you meet more friends or connect with organizations and groups?
d5	How would you feel if someone could accompany you in doing things you enjoy?
d6	How would you feel if someone could provide a platform for you to showcase or develop your interests and hobbies?

## Discussion

With the accelerating trend of population aging and the continued promotion of home-based care of older adults, the demand for informal care at the community level has become increasingly prominent [41]. A European study revealed that 40.0% of older adults reported not receiving timely assistance, and for nearly all of them, the family was the only source of support, with few reporting the use of other community service resources [42]. A scientifically sound and systematic assessment tool is essential for accurately identifying the assistance needs of home-dwelling older adults and enhancing the efficiency of resource allocation. However, existing assessment tools for older adults’ service needs [14–19] primarily focus on medical care, functional support, or unmet needs. To date, no dedicated tool has been developed to evaluate the unpaid assistance needs specific to community-dwelling older adults.

To fill this research gap, the present study developed and validated a psychometrically robust assistance needs assessment scale. The scale consists of 27 concise and well-structured items. With a completion time of approximately 10 min, the content is accessible and easy to understand, making it suitable for older adults with varying levels of education. Covering multiple dimensions—including daily living assistance, health maintenance, emotional support, and social participation—the scale accommodates the needs of healthy, sub-healthy, and partially functionally limited older adults, aligning well with the growing demand for personalized, multi-level care services of older adults. The scale was developed through a comprehensive multi-phase process involving literature review, qualitative interviews, expert consultation, pilot testing, and quantitative validation [43, 44]. This ensured the multidimensional nature of assistance needs was thoroughly reflected. The results demonstrated strong reliability, content validity, and structural validity, indicating stable and interpretable psychometric properties.

Notably, this scale distinguishes itself from existing tools by focusing not only on unmet or general care needs of older adults but also on older adults’ expectations regarding socially engaged caregiving. It emphasizes the value of informal support networks and highlights the importance of participation, autonomy, and emotional well-being in later life. The tool offers a scientific foundation for community health workers, healthcare professionals, and volunteer organizations to assess needs and allocate support resources more effectively through stratified interventions [45, 46]. Furthermore, this instrument provides essential data for evaluating and improving care policies of older adults under the aging population context. Its application supports the optimization of community support systems, improves responsiveness, and reduces the duplication or misallocation of public welfare resources. Ultimately, it contributes to enhancing older adults’ sense of support, well-being, and security in home-based settings.

The development of this scale strictly followed standard procedures and principles for scale construction. First, relevant domestic and international literature and books were reviewed, and findings from the previous phase of qualitative research were integrated. Based on this, a preliminary structure and content of the scale were drafted through group discussions to ensure both the breadth and depth of the item pool. To enhance the scientific rigor, accuracy, comprehensiveness, and operational feasibility of the items, two rounds of expert consultation were conducted to refine and improve the scale. A pilot survey was then used to optimize item wording for better readability and to identify potential problems and challenges in the implementation process, thereby ensuring the feasibility of the formal study. After completing the formal survey, item analysis, reliability analysis, validity analysis, and acceptability analysis were conducted to further refine and finalize the scale.

Throughout the scale development process, quality control was implemented at every stage by the research team and supervisors. The expert consultation was carried out strictly in accordance with established procedures. All participating experts held at least a bachelor’s degree and a senior professional title (associate professor or above), with an average of more than 27 years of work experience, indicating high credibility. The experts came from multiple provinces and research fields, demonstrating broad representativeness. The valid response rates for the two rounds of expert consultation were 88.2% and 100.0%, significantly exceeding the 70% benchmark, indicating a high level of expert engagement. The authority coefficient of the experts was 0.86, reflecting a high degree of expertise and credibility. A total of 31 suggestions were collected in the first round of consultation, and 6 suggestions in the second round, indicating a convergence of expert opinions. After two rounds, the mean importance scores for all items were above 4.00, suggesting high consensus among experts. The coefficient of variation for all items was below 0.25, indicating strong agreement. The item-level content validity index (I-CVI) for all items exceeded 0.78, and the scale-level content validity index (S-CVI) was above 0.90, demonstrating good content validity. All items showed item-total correlation coefficients above 0.40, indicating high internal consistency. The overall Cronbach’s alpha coefficient of the scale was 0.974, confirming excellent internal reliability. Bartlett’s test of sphericity indicated that the scale was suitable for factor analysis. After further refinement and item screening, confirmatory factor analysis showed good model fit with the following indices: CFI = 0.971, TLI = 0.968, RMSEA = 0.053, CMIN/DF = 1.580, NFI = 0.926, and IFI = 0.972, indicating the scale also possesses strong structural validity. furthermore, preliminary evidence of predictive validity was found, with scale scores showing a moderate positive correlation with depressive symptoms and a weak negative correlation with activities of daily living. These findings align with theoretical expectations, suggesting that the scale reflects important psychosocial and functional aspects of older adults and may help identify vulnerable groups. Future longitudinal studies are needed to further confirm its predictive value for outcomes such as hospitalization, social isolation, or caregiver burden.

In addition to its policy implications, the findings can be better understood when viewed through established theoretical perspectives on aging. The principle of aging in place emphasizes supporting older adults to remain safely and independently in their familiar communities, which has become a central goal of aging policy worldwide [47]. By identifying unmet assistance needs within home and community settings, the scale provides practical evidence to inform strategies that promote aging in place. From the perspective of social capital, strong community ties and reciprocal support are essential for maintaining well-being and reducing isolation among older adults [48]. The scale’s focus on unpaid assistance directly reflects this dimension, highlighting the importance of mobilizing community and volunteer resources as social capital to improve care. Finally, caregiving frameworks emphasize that care involves not only physical support but also psychosocial and emotional aspects [49]. By capturing both functional and psychosocial needs, the scale contributes to more comprehensive caregiving approaches that integrate family, community, and volunteer systems.

### Limitation

This study has several limitations. First, convenience sampling may have introduced selection bias and affected sample representativeness. Second, participants were recruited exclusively from urban communities in central China, which may limit the generalizability of the findings to rural or economically disadvantaged regions where assistance needs could differ substantially. Future studies should expand the geographic scope and adopt more representative sampling strategies to improve external validity. Third, since most of the supporting literature and validation data are derived from Chinese contexts, the cross-national generalizability of the scale may be limited. Replication and validation in different cultural and healthcare settings are needed to establish its broader applicability. Fourth, the study excluded high-risk groups such as the functionally impaired, socially isolated, or low-income older adults living alone, limiting the scale’s applicability in these populations. Lastly, while the scale underwent expert consultation, pilot testing, and psychometric validation, its predictive validity and long-term utility require further large-scale and longitudinal studies.

## Conclusion

This study followed a standardized scale development process, integrating literature review, qualitative research, Delphi expert consultation, and a formal questionnaire survey to develop and validate a 27-item assessment tool for evaluating the assistance needs of home-dwelling older adults. The scale demonstrated strong scientific rigor, reliability, validity, and practical applicability. The finalized version comprises four dimensions: life assistance, health maintenance, visiting and communication, and social interaction. The results indicated that the scale has good reliability and validity. This tool is a reliable and effective instrument for assessing unpaid assistance needs among older adults living at home and provides a scientific basis for the planning, allocation, and optimization of community-based care services of older adults and related policy development.

## Data Availability

I declare that all data and materials are available from the corresponding author upon reasonable request.

## Appendix 1

**Table 5:** Interview guide

1. In your daily life, what types of assistance would you like to receive? Could you elaborate? 2. What specific expectations or hopes do you have regarding receiving assistance? In what aspects would you like the helper to improve? 3. In what form would you prefer volunteers to assist you? Why do you favor this form? 4. In your opinion, how should helpers provide services to bring the greatest benefit to older adults? 5. Do you have anything else you would like to add regarding this interview?	

## Appendix 2

**Table 6:** Basic information of consulted experts (*n*=15)

Item	Category	*n*	%
Gender	Male	2	13.3
	Female	13	86.7
Education	Bachelor’s	7	46.7
	Master’s	5	33.3
	Doctorate	3	20.0
Professional Title	Associate Senior	7	46.7
	Full Senior	8	53.3
Field of Expertise	Geriatric Nursing	3	20.0
	Community Nursing	2	13.3
	Clinical Nursing	4	26.7
	Nursing Management	3	20.0
	Psychological Nursing	1	6.7
	Social Security	2	13.3

## Appendix 3

**Table 7:** Item pool of the assistance needs assessment scale for home-dwelling older adults

Dimension	Items
Daily Care	a1 If someone could help you with grocery shopping and cooking, how would you feel?
	a2 If someone could deliver meals that meet your nutritional needs and taste good, how would you feel?
	a3 If a volunteer could help you maintain personal hygiene (e.g., bathing, changing clothes), how would you feel?
	a4 If a volunteer could assist you in moving around your room, how would you feel?
	a5 If a volunteer could accompany you for a walk outdoors, how would you feel?
	a6 If a volunteer could help you clean your home, how would you feel?
	a7 If a volunteer could provide help such as home repairs, indoor lighting adjustment, or safety checks, how would you feel?
	a8 If a volunteer could proactively ask about difficulties in your daily life and try to help solve them, how would you feel?
	a9 If a volunteer could provide material support for your daily necessities, how would you feel?
Medical Care	b1 If a volunteer could help you with physical checkups, how would you feel?
	b2 If a volunteer could guide you in medication use and self-care, how would you feel?
	b3 If a volunteer could teach you about health maintenance and disease prevention, how would you feel?
	b4 If a volunteer could help you take preventive measures against falls or other injuries, how would you feel?
	b5 If a volunteer could accompany you to hospital visits or health checkups, how would you feel?
	b6 If a volunteer could come promptly and offer assistance when you feel unwell, how would you feel?
	b7 If a volunteer could guide and accompany you in doing rehabilitation or physical exercises, how would you feel?
	b8 If a volunteer could help you consult medical experts about your health problems, how would you feel?
	b9 If a volunteer could visit you when you are ill, how would you feel?
	b10 If a volunteer could stay with you when you are ill, how would you feel?
Psychological Comfort	c1 If a volunteer could chat with you, how would you feel?
	c2 If a volunteer could listen to you when you are upset, how would you feel?
	c3 If a volunteer could encourage your non-cohabiting family members to visit you, how would you feel?
	c4 If a volunteer could encourage your friends, colleagues, or neighbors to visit you, how would you feel?
	c5 If a volunteer could encourage your relatives to visit you, how would you feel?
	c6 If a volunteer could celebrate your birthday with you, how would you feel?
	c7 If a volunteer could detect your negative emotions while chatting and give reasonable advice, how would you feel?
	c8 If a volunteer could patiently listen to your troubles and provide support or help, how would you feel?
	c9 If a volunteer could respect and appreciate you, how would you feel?
	c10 If a volunteer could care about your family and help address your worries about their difficulties, how would you feel?
Cultural and Recreational Engagement	d1 If a volunteer could teach you how to use mobile phones and the internet to enrich your life and increase enjoyment, how would you feel?
	d2 If a volunteer could accompany you in activities you are interested in, how would you feel?
	d3 If a volunteer could praise you and acknowledge your achievements, how would you feel?
	d4 If a volunteer could accompany you in doing things you enjoy, such as dining out or sightseeing, how would you feel?
Social Interaction	e1 If a volunteer could register or contact you to participate in social activities, how would you feel?
	e2 If a volunteer could accompany you in attending social activities, how would you feel?
	e3 If a volunteer could organize social or group activities for older adults, how would you feel?
	e4 If a volunteer could help you make new friends or connect with social groups or organizations, how would you feel?

## Appendix 4

**Table 8:** Round 1 expert ratings on item importance

Item	Mean Score	Coefficient of Variation	Item	Mean Score	Coefficient of Variation	Item	Mean Score	Coefficient of Variation
a1	4.53	0.16	b5	4.60	0.14	c8	4.60	0.16
a2	4.33	0.19	b6	4.60	0.14	c9	4.40	0.17
a3	4.33	0.17	b7	4.53	0.16	c10	4.20	0.23
a4	4.33	0.17	b8	4.47	0.14	d1	4.33	0.17
a5	4.20	0.18	b9	4.33	0.17	d2	4.40	0.17
a6	4.13	0.15	b10	4.60	0.16	d3	4.47	0.17
a7	4.33	0.17	c1	4.40	0.17	d4	4.47	0.17
a8	4.53	0.14	c2	4.60	0.14	e1	4.27	0.19
a9	4.20	0.21	c3	4.40	0.21	e2	4.47	0.14
b1	4.60	0.14	c4	4.47	0.17	e3	4.33	0.17
b2	4.60	0.14	c5	4.40	0.19	e4	4.33	0.14
b3	4.47	0.17	c6	4.27	0.19			
b4	4.60	0.14	c7	4.53	0.14			

## Appendix 5

**Table 9:** Round 2 expert ratings on item importance

Item	Mean Score	Coefficient of Variation	Item	Mean Score	Coefficient of Variation	Item	Mean Score	Coefficient of Variation
a1	4.53	0.16	b2	4.87	0.07	c4	4.53	0.14
a2	4.53	0.16	b3	4.73	0.13	c5	4.47	0.14
a3	4.53	0.16	b4	4.87	0.07	c6	4.80	0.09
a4	4.40	0.21	b5	4.80	0.09	c7	4.73	0.13
a5	4.40	0.21	b6	4.93	0.05	c8	4.67	0.13
a6	4.53	0.16	b7	4.73	0.10	d1	4.00	0.23
a7	4.67	0.13	b8	4.67	0.10	d2	4.33	0.14
a8	4.73	0.10	b9	4.80	0.09	d3	4.00	0.19
a9	4.67	0.10	c1	4.67	0.13	d4	4.00	0.19
a10	4.67	0.13	c2	4.60	0.14	d5	4.27	0.16
b1	4.87	0.07	c3	4.53	0.16	d6	4.67	0.13

## Appendix 6

**Table 10:** Round 1 expert ratings on content validity

Item	I-CVI	S-CVI	Item	I-CVI	S-CVI	Item	I-CVI	S-CVI
a1	0.87	0.84	b5	0.93	0.84	c8	0.73	0.84
a2	0.87		b6	0.93		c9	0.80	
a3	0.87		b7	0.93		c10	0.80	
a4	0.80		b8	0.73		d1	0.87	
a5	0.80		b9	0.93		d2	0.87	
a6	0.73		b10	0.87		d3	0.93	
a7	0.73		c1	0.93		d4	0.73	
a8	0.73		c2	0.73		e1	0.73	
a9	0.80		c3	0.87		e2	0.87	
b1	0.93		c4	0.93		e3	0.87	
b2	0.87		c5	0.47		e4	0.73	
b3	0.93		c6	0.93				
b4	0.93		c7	0.93				

## Appendix 7

**Table 11:** Round 2 expert ratings on content validity

Item	I-CVI	S-CVI	Item	I-CVI	S-CVI	Item	I-CVI	S-CVI
a1	0.93	0.96	b2	1.00	0.96	c4	1.00	0.96
a2	0.93		b3	1.00		c5	1.00	
a3	0.87		b4	1.00		c6	1.00	
a4	0.87		b5	1.00		c7	0.93	
a5	0.87		b6	1.00		c8	0.93	
a6	0.93		b7	1.00		d1	0.80	
a7	1.00		b8	1.00		d2	1.00	
a8	1.00		b9	1.00		d3	0.93	
a9	1.00		c1	1.00		d4	0.87	
a10	1.00		c2	1.00		d5	1.00	
b1	1.00		c3	1.00		d6	0.93	

## Appendix 8

**Table 12:** Summary of revision suggestions

1) Round 1 Expert Consultation A total of 31 suggestions were collected in the first round, of which 25 were adopted or thoroughly considered. Based on expert feedback, corresponding deletions, modifications, or additions were made to the items. The revision process is summarized as follows: ① Daily Care Dimension For item a1, one expert suggested deleting the phrase "cooking." After discussion, this suggestion was adopted. For item a2, one expert proposed revising "meals that meet nutritional needs and taste good." The content was improved accordingly. For item a3, one expert suggested adding "body wiping." The suggestion was adopted. For item a6, an expert recommended changing “cleaning the house” to “doing household cleaning.” The revision was made. For item a7, the expert advised revising “providing help such as home repairs, adjusting indoor lighting, and checking for safety hazards” to “coming to your home to assist, such as adjusting lighting or checking for safety hazards.” The suggestion was adopted. For item a8, one expert recommended deletion. The team agreed and removed it. For item a9, a revision was made from “providing material support for daily necessities” to “delivering daily necessities.” One expert suggested adding a new item: “Assisting with financial matters such as bank interest and deposits.” The suggestion was adopted. ② Medical Care Dimension For item b1, the wording was changed from “helping with physical checkups” to “providing checkups such as blood pressure and blood glucose monitoring.” Items b2–b4 were revised based on one expert's suggestions. For item b8, deletion was suggested and adopted, considering its I-CVI did not meet the criteria. One expert proposed adding “early detection after events such as falls may be more important.” Given the existing item b6 (“responding and assisting promptly when you feel unwell”) covers this, a new item was not added. However, b6 was revised to “responding and assisting promptly when you seek help due to physical discomfort.” For items b9 and b10, the phrase “when you are ill” was changed to “after learning that you are ill.” Item b8 was again recommended for deletion and removed accordingly. ③ Psychological Comfort Dimension For items c1 and c2, merging was recommended. Given that c2 also had an inadequate I-CVI, it was deleted. For item c3, the phrase “encourage non-cohabiting family to visit” was revised to “try to contact non-cohabiting family to visit.” For item c5, two experts suggested deletion. Since its I-CVI was also insufficient, it was deleted. However, to preserve content, c4 was revised. For items c7 and c8, merging was suggested. Given c8’s low I-CVI, it was deleted. One expert proposed merging c9 and d3. However, due to recurring mentions in qualitative interviews and satisfactory metrics, both were retained with revisions to avoid redundancy. ④ Cultural Engagement Dimension Items d2 and d4 were recommended for merging. Considering the low I-CVI of d4, it was deleted and d2 revised. ⑤ Social Interaction Dimension One expert suggested adding: “If volunteers can provide you with a platform to showcase or develop hobbies, how would you feel?” The suggestion was adopted. Additionally, one expert recommended reorganizing the five dimensions into four: “Daily Assistance, Health Maintenance, Visiting and Communication, Social Interaction.” This was adopted. d1 was reclassified under “Daily Assistance” d2 under “Social Interaction” d3 under “Visiting and Communication” 2) Round 2 Expert Consultation A total of 6 suggestions were collected, all of which were either adopted or thoroughly considered. Revisions were made as follows: ① Daily Assistance Dimension For item a3, the phrase “maintain personal hygiene such as wiping the body, bathing, or changing clothes” was changed to “wiping the body, bathing, or changing clothes.” ② Health Maintenance Dimension For item b1, one expert recommended adding “at home,” which was adopted. For item b4, the phrase “providing fall prevention guidance” was revised to “explaining how to prevent falls.” ③ Visiting and Communication Dimension For item c7, the phrase “feel respected and appreciated” was revised to “feel respected and appreciated when receiving help.” Additionally: One expert suggested renaming the dimension “Daily Assistance” to “Daily Support.” This was adopted. One expert recommended adding “irregular provision of services” to all items for rigor. The suggestion was adopted and applied across all items. Conclusion: After two rounds of expert consultation, the preliminary draft of the Urban Home-Dwelling Older Adults Volunteer Service Needs Assessment Scale was finalized. It includes four dimensions: Daily Support (10 items) Health Maintenance (9 items) Visiting and Communication (8 items) Social Interaction (6 items) Total: 33 items.	

## Appendix 9

**Table 13:** Basic information of participants (*n* = 380)

Variable	Value
Age [years, M (P25, P75)]	72（66, 80）
Gender	
Male	150（39.5）
Female	230（60.5）
Education level	
Primary school or below	150（39.5）
Junior high schoo	100（26.3）
Senior high school or above	130（34.2）
Religious belief	
Yes	32（8.4）
No	348（91.6）
Previous occupation	
Government employee	75（19.7）
Worker	129（33.9）
Farmer	91（23.9）
Other	85（22.4）
Marital status	
Married	255（67.1）
Other*	125（32.9）
Living alone	
Yes	70（18.4）
No	310（81.6）
Without caregiver	
Yes	261（68.7）
No	119（31.3）
Pension	
Yes	289（76.1）
No	91（23.9）
Financial support from relatives/friends	
Yes	8（2.1）
No	372（97.9）
Government subsidy	
Yes	42（11.1）
No	338（88.9）

## Appendix 10

**Table 14:** Overall reliability analysis of the scale

Item	Corrected Item-Total Correlation	Cronbach’s Alpha if Item Deleted	Item	Corrected Item-Total Correlation	Cronbach’s Alpha if Item Deleted
The Cronbach’s alpha coefficient for the overall scale is 0.974					
a1	0.772	0.973	b8	0.751	0.973
a2	0.743	0.973	b9	0.756	0.973
a3	0.610	0.974	c1	0.789	0.973
a4	0.599	0.974	c2	0.741	0.973
a5	0.806	0.973	c3	0.745	0.973
a6	0.779	0.973	c4	0.728	0.973
a7	0.731	0.973	c5	0.718	0.973
a8	0.852	0.973	c6	0.747	0.973
a9	0.754	0.973	c7	0.661	0.974
a10	0.677	0.974	c8	0.760	0.973
b1	0.729	0.973	d1	0.540	0.974
b2	0.724	0.973	d2	0.523	0.974
b3	0.748	0.973	d3	0.524	0.974
b4	0.776	0.973	d4	0.542	0.974
b5	0.801	0.973	d5	0.568	0.974
b6	0.704	0.973	d6	0.614	0.974
b7	0.789	0.973			

## Appendix 11

**Table 15:** Reliability analysis by dimension

Item	Corrected Item-Total Correlation	Cronbach’s Alpha if Item Deleted	Item	Corrected Item-Total Correlation	Cronbach’s Alpha if Item Deleted
The Cronbach’s alpha coefficient for the Daily Support dimension is 0.954			b8	0.751	0.965
a1	0.840	0.947	b9	0.756	0.966
a2	0.805	0.948	The Cronbach’s alpha coefficient for the Visiting and Communication dimension is 0.954		
a3	0.722	0.951	c1	0.797	0.948
a4	0.709	0.951	c2	0.797	0.950
a5	0.847	0.946	c3	0.783	0.950
a6	0.830	0.947	c4	0.799	0.947
a7	0.782	0.949	c5	0.775	0.951
a8	0.886	0.945	c6	0.849	0.945
a9	0.759	0.950	c7	0.767	0.949
a10	0.735	0.951	c8	0.858	0.945

The Cronbach’s alpha coefficient for the Health Maintenance dimension is 0.967			The Cronbach’s alpha coefficient for the Social Interaction dimension is 0.958		
b1	0.825	0.963	d1	0.873	0.948
b2	0.853	0.962	d2	0.878	0.946
b3	0.847	0.962	d3	0.878	0.946
b4	0.878	0.961	d4	0.884	0.950
b5	0.887	0.961	d5	0.748	0.958
b6	0.704	0.965	d6	0.849	0.949
b7	0.789	0.962			
